# Facilitating Green Supply Chain in Dental Care through Kansei Healthscape of Positive Emotions

**DOI:** 10.3390/ijerph16193507

**Published:** 2019-09-20

**Authors:** Ling-Hsin Hsu, Yu-Hsiang Hsiao

**Affiliations:** 1 Department of Business Administration, National Taipei University, New Taipei City 23741, Taiwan; 2 Department of Dentistry, Taipei City Hospital, Taipei City 10078, Taiwan

**Keywords:** green supply chain, dental healthscape, positive emotions, Kansei engineering, stimulus–organism–response model

## Abstract

Dentistry is highly energy- and resource-intensive with a significant environmental impact. To consolidate green dentistry supply chains, delivering the care of highest quality that meets client value should not be neglected. This study emphasized the importance of client-centered healthscape design for facilitating a green dentistry supply chain. A client-centered healthscape design, which promotes clients’ positive emotions and increases willingness to revisit the dentist, plays a critical role in realizing green dentistry supply chains in the long run. For this purpose, the relationship among dental healthscape design elements, client emotions, and revisit intentions was investigated using a Kansei engineering-based approach. The effects of dental healthscape elements on clients’ positive emotions and the effects of positive emotions on clients’ revisit intentions were holistically examined on the basis of the stimulus–organism–response model. Through this approach, 17 elements of design, ambience, and social interaction factors that comprise the dental healthscape and 20 Kansei words used to express clients’ positive emotions regarding dental service were identified. A questionnaire survey was used to assess Kansei and revisit intention in healthscape scenarios, composed of varied design elements. Primary data were collected from 600 individuals from 2017 to 2018 throughout Taiwan. Partial least squares was applied to holistically analyze the effects of dental healthscape elements on clients’ positive emotions and the effects of positive emotions on clients’ revisit intention to generate a Kansei model for the dental healthscape. All 20 Kansei words had significant positive effects on the dental revisit intention of clients. The five positive emotions most associated with increased revisit intention were *thoughtful*, *hopeful*, *tender*, *comfortable*, and *cozy*. The Kansei model of the dental healthscape provides references for healthscape design that maintains positive client emotions during the dental service and results in high revisit intention. This approach can realize an emotion-centered design for dental healthscapes that promotes preventive dental care, early treatment, and effective use of medical resources, and consequently contributes to green dentistry supply chains.

## 1. Introduction

A supply chain is a system of organizations, people, information, and resources involved directly or indirectly in different activities that produce value in the form of physical goods or services delivered from supplier to the ultimate consumer [1]. Services, unlike physical goods, are intangible, perishable, and heterogeneous, and often involve customers as active participants in the production process. The implication is that all services have customers as primary suppliers of inputs, which is the customer–supplier duality [2]. Converting or creating value for customers in services always involves the customers actively; customers are often co-producers. Customer-introduced variability makes service supply chain management complicated and difficult [3]. The main goals of supply chain management can be explained, such as increasing customer satisfaction, decreasing the cycle time, decreasing the storage costs, decreasing the product defects, and decreasing the activity costs. Green supply chain management aims to achieve these goals by integrating environmentalist approaches which minimize or eliminate wastage, including hazardous chemicals, emissions, energy, and solid waste along the supply chain [4,5,6]. As such, green supply chain management plays a vital role in influencing the total environmental impact of any element involved in supply chain activities and thus contributes to sustainability performance enhancement [7].

**Figure 1:**
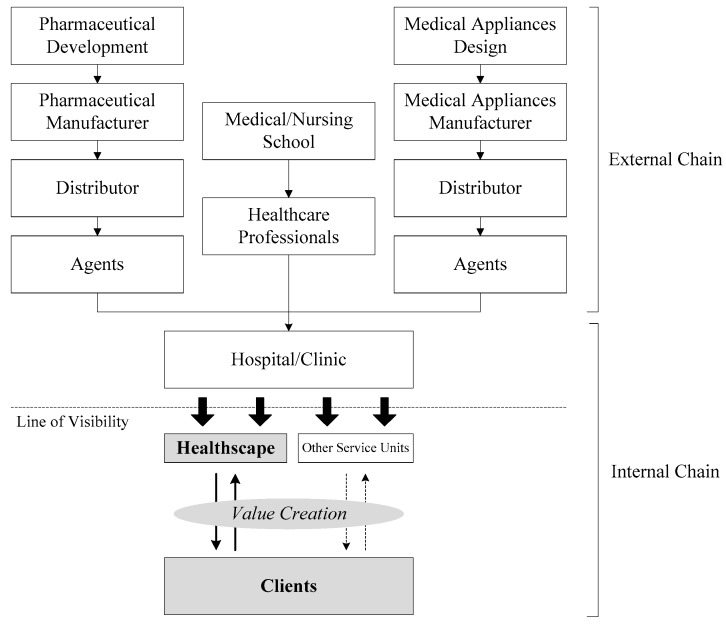
Healthcare supply chain structure.

Healthcare, a division of the service sector, is one of the largest contributors to waste production in the world. Given increased awareness of the environmental costs associated with waste disposal and its public health impact, many healthcare institutions are adopting environmentally friendly “green” practices that reduce waste production and offer equally effective, yet less expensive alternatives [8]. Figure 1 shows the healthcare supply chain structure, which includes external chain and internal chain. The external chain comprises three sub-chains of providing pharmaceuticals, medical appliances, and healthcare professionals that support the healthcare service processes in the internal chain. To achieve a green status in healthcare supply chains, practices such as reducing waste, protecting the environment, and making good use of resources have been implemented in the process of external and internal chains. More proactively, eco-design, which aims to avoid environmental damage beforehand instead of reducing waste afterwards by actively making environmentally friendly decisions in the product design phase [9], has attracted a lot of attention in recent years. Eco-design considers both environmental and economic aspects associated with the life cycle of products and processes without compromising performance, functionality, and quality [9,10]. Various relevant literature and practical implementations have followed the guideline of eco-design [9] in the development of green healthcare buildings and facilities, low-energy equipment, medical equipment and material recycling, low environmental hazard drugs, low emission of greenhouse gases, efficient healthcare logistics, and so on [6,7,8,11,12,13,14,15,16]. However, these implementations were derived from the perspective of "physical goods". As a service, of which the process itself is the product, patients’ or clients’ perceived value of the healthcare service is mostly generated during their receiving of it. Therefore, in pursuit of effective value of healthcare supply chain management, the interface of healthcare service process for interacting and contacting with patients or clients plays a critical role. Figure 1 highlights the importance of interaction between clients and hospitals/clinics, which creates the core value of healthcare services in a healthcare supply chain structure. Aside from this, if the healthcare service cannot be designed for patients or clients in terms of medical and psychological needs, or the patient- or client-introduced variability makes the service process unsuccessful, it may cost additional medical materials and energy, and generate more ineffective medical practices and waste. Also, it causes the supply chain activities that are prepared for the healthcare service to lose their value, violating the pursuit of green supply chains in healthcare. Scholars suggested defining patients as clients, and in order to provide healthcare services, clients, doctors, and providers have to participate in the discussion of treatment planning and decision-making. This value co-creation behavior contributes to the supply chain management in the healthcare system [17,18]. Improving patient or client value is the basis for green healthcare supply chains. Moreover, enhancing the “first-time yield” is the best way to reduce waste and use resources efficiently.

Dentistry is highly energy- and resource-intensive with a significant environmental impact. Factors inherent in the profession, such as enormous electricity demands of electronic dental equipment, voluminous water requirements, environmental effects of biomaterials (before, during, and after clinical use), the use of radiation, and the generation of hazardous waste all contribute towards this [8]. Adoption of eco-design practices [9] and environmentally friendly measures has been emphasized and studied in the literature [8,12,13]. To consolidate green dentistry supply chains, delivering the care of highest quality which meets client value should not be neglected. For this purpose, the element of positive experience, which is formed by customizing treatment to client needs, and the element of safe care, by which clients are protected from physical, psychological, or emotional harm, should be added in the building of green clinical care [8].

Oral condition greatly affects physical health, mental health, and quality of life [19,20,21]. Barriers to dental care for many people include anxiety, cost considerations, perceived needs, and lack of access [22]. In Taiwan, since the implementation of national health insurance in 1995, citizens have had a high-quality, easily accessible, and low-expense medical care system. Although the promotion of preventive care, early treatment, and regular use of dental services have become easier, eliminating clients’ anxiety and fear remains a challenge [23,24,25,26]. However, this strongly affects clients’ perceived value. The main reason for people to visit the dentist is to relieve oral discomfort, but many people delay treatment when experiencing fear and anxiety [27,28]. This worsens the oral condition, increases the treatment complexity and time, and decreases ultimate patient satisfaction [23,29]. Consequently, more resources, chemicals, and drugs will be used, and waste will be generated, all of which are indirectly harmful to the environment.

Fear, as defined in the Cambridge dictionary, is an unpleasant emotion or thought that people have when they contemplate something potentially dangerous or painful. Anxiety is an uncomfortable feeling of nervousness about something that is happening or may happen, or it may be defined as an adverse or irrational reaction to an unknown future [30]. The fear of painful experiences may exacerbate the anxiety of visiting the dentist [31,32]. Research discovered that people with a high level of conscious anxiety appeared to be more susceptible to pain before, during, and after dental treatment [28]. Excessive negative emotional responses to specific procedures or the environment of dental treatment have made people reluctant to use dental services [33,34].

Emotions are generated when an individual assesses the personal meaning of some antecedent event, environment, relationship, or encounter. This appraisal process may be either conscious or unconscious, and it triggers a series of reactional tendencies, such as subjective experience, cognitive processing, and physiological changes [35]. Emotions play a significant role in promoting customer satisfaction and loyalty, both in product and service experience [36]. Negative emotions, such as fear and anxiety, lead to specific fight-or-flight responses, making an individual’s thought–action repertoires narrower than those in a neutral state [35]. By contrast, the broaden-and-build theory of positive emotions suggests that positive emotions can broaden an individual’s awareness; encourage novel, varied, and exploratory thoughts and actions; help build lasting personal resources; and generate indirect and long-term adaptive benefits [37]. Research has demonstrated that positive emotions can improve parasympathetic cardiac control, benefit physical health [38], distract people from their pain, extend past happiness, and build energy to face threats and uncertainties [39,40]. Fear leads to escapism and anger that may provoke attacks, but positive emotional responses can lead to positive evaluations of a clinical experience [41]. Positive emotions can transform negative memories produced by bad experiences, expand thought, and create a positive experience that can extend to future visits, in a clinical context [39]. On the basis of the broaden-and-build theory of positive emotions, maintaining or arousing clients’ positive emotions can not only increase their willingness to share information and actively participate in treatment, but also enhance their experience and improve their satisfaction [39,40,42,43]. 

Dental care is highly customized, and individual clinical consultations are usually longer than those in other medical fields [44]. During the course of diagnosis and treatment, patients have more opportunities to interact with and be affected by their perceptions of the dental healthscape. Correspondingly, the dental healthscape provides opportunities to improve patients’ impressions of the dental treatment process and reduce patients’ negative emotions. Kotler [45] believes that the “atmospherics” can be used to define the service environment space, wherein it is carefully designed so that a consumer who is in it can get a special emotional feeling. Originating from the concept of the servicescape, the healthscape is defined as the physical environment of healthcare services and refers to the tangibles experienced through senses such as sight, smell, sound, taste, and touch; the healthscape is believed to affect client perceptions, attitudes, satisfaction, and behavior [46,47,48,49]. To justify the relationship among dental healthscape, clients’ emotions and behavior, the stimulus-organism-response (SOR) model [50] can be the theoretical foundation. The SOR model suggests that the environment stimulates the organism, and the organism, accepting such stimuli, produces through an internal process the final reactions of approach or avoidance [50]. Many studies have focused on the effects of the servicescape on consumer emotions and perceptions toward services, mainly hedonic services [51,52,53,54,55]. The servicescape affects an individual’s emotional response, which, in turn, drives the individual to react in various ways, and similar findings have been reported for the healthscape. Pai and Chary [47] discovered that in a teaching hospital in India, the healthscape had a stronger influence on patients’ behavioral intention than did overall service quality. Hermawan and Yusran [46] observed that atmospheric factors of dental clinics in Indonesia positively influenced patients’ emotions and behavioral intentions. Sag et al. [49] reviewed the limited literature on the servicescape in healthcare settings and indicated significant positive associations between the healthscape and patient perceptions, satisfaction, and emotions. However, the study of the effects of the dental healthscape on clients’ emotions and behavior is limited. Healthscape design can drive creation of positive client emotions during dental care services. Healthscape design is highly influential, but it has rarely been the focus of research [56]. Lin and Mattila [53] suggested that, following the concept of Gestalt, customers generally receive a variety of stimuli from the restaurant servicescape and view every encounter holistically, considering multiple aspects in their experience evaluation. Customer experience is holistic, encompassing every contact with a firm, so all service encounters need to be seamlessly orchestrated [57]. Accordingly, this study assumed clients to consider multiple atmospheric cues, tangible elements, and services of the healthscape. A comprehensive study of the dental healthscape’s effect on clients’ emotions and consequent behavior is required and will contribute to the dental care field.

Negative memories arouse uneasiness and anxiety [27]. When entering a dental clinic, people’s emotions can be magnified or attenuated by the atmospherics of the healthscape. Client involvement should be a primary concern in the design of dental service processes, and all care practices should incorporate emotional factors, rather than focusing solely on technical compliance [58]. However, producing this synergy is challenging. The design of medical services varies from that of a manufactured product; medical service design focuses on not only the design of the service itself but the combined effects of the care elements and delivery processes. A client’s perception of service design is a complex system [58]. Although methods, such as quality function development, the Kano model, and conjoint analysis, can help understanding customers’ explicit needs, none of these adequately translate the effect of or implicit need for design parameters [59]. Kansei engineering (KE), an ergonomic methodology for consumer emotion-oriented design, analyzes consumer psychology and translates the results into product development [60]. As part of the Eastern psychological vocabulary, *Kansei* represents factors such as needs, wants, preferences, emotions, affections, and feelings. KE has been applied in various circumstances, including to the industrial design of cars, refrigerators, kitchen systems, toilets, and mattresses [61]; tourism service design [62]; logistics [63]; and airlines [64]. An emotion-oriented design of the dental healthscape may play a crucial role in reducing a client’s anxiety during the care process and leaving a positive impression, which promotes future willingness to visit. KE advances the understanding of the individual and the integrated effects of healthscape design elements on clients’ Kansei perception. 

**Figure 2:**
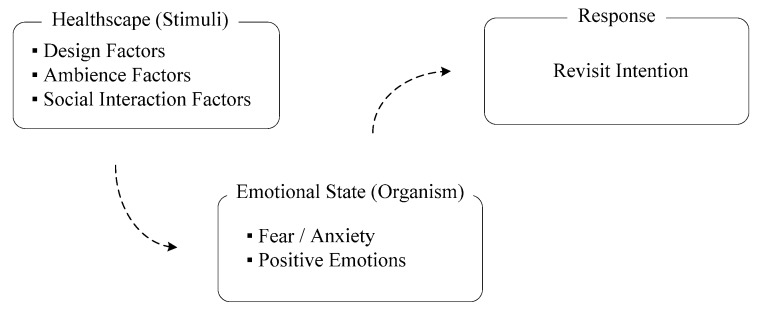
Modified stimulus-organism-response (SOR) model.

A positive atmosphere in a healthscape is consequential because it arouses people’s positive emotions and encourages dental visits for regular oral examinations, thus promoting oral health. In this study, the assumed causal links between the dental healthscape and clients’ emotions and revisit intentions were based on the SOR model, as illustrated in Figure 2. The three dimensions of the servicescape proposed by Hightower [65]—the dimensions of the design, ambience, and social interaction—were selected to define the design properties of a dental healthscape. Clients view every encounter holistically and consider multiple aspects in their experiences and emotional evaluations [53,57]. These evaluations are the psychological responses of clients to the combined outcomes of all properties of the healthscape. Studying the effects of a single property without considering others is insufficient. KE is an appropriate methodology for the comprehensive study of the main and combined effects of multiple properties. The KE methodology was used to analyze the effects of dental healthscape design properties on clients’ emotional and behavioral responses. The purposes of this study were to firstly identify a comprehensive set of positive Kansei that will attract clients to dental services; secondly, to identify the healthscape elements of the dental service environment and analyze their effects on clients’ Kansei; and thirdly, analyze the effects of clients’ Kansei on their revisit intention. The results are intended to provide knowledge regarding which Kansei most positively affects client revisit intention and to indicate which healthscape design elements create these notable Kansei for the dental service environment. This knowledge can contribute to delivering the care of highest quality which meets client values, thus consolidating green dentistry supply chains.

## 2. Methods

KE is a method to convert customers’ ambiguous impressions about products into a detailed product design and to provide designers with guidance for product development in accordance with customers’ Kansei. KE helps customers choose from a variety of designs that fit their Kansei. The methodology of KE includes a psychological evaluation experiment followed by multivariate analyses. According to Schutte et al. [66], implementation of KE begins with determining the research domain, which includes defining the intended target group, user type, market niche, and product group. The second step spans the Kansei semantic space, a collection of numerous Kansei words or expressions to describe the specific domain. The third step spans the property space by identifying product properties that represent the domain and selecting key features for further evaluation. In the fourth step, Kansei evaluation experiments are conducted to synthesize semantic and property spaces. This is commonly implemented through customer perception surveys in which customers evaluate product examples composed of key features in the property space using all of the Kansei words in the semantic space. Finally, the effects of the product features on the Kansei are determined from the survey data. Once the effects are validated, a recommendation model is generated to guide the Kansei design of a particular product.

**Figure 3:**
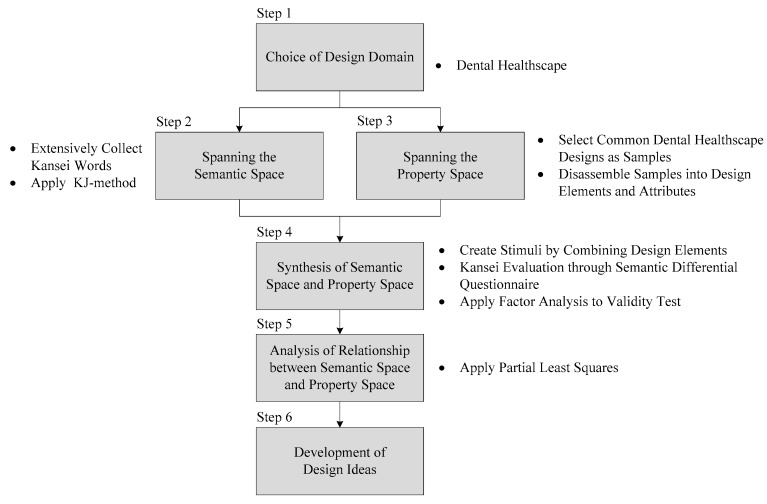
Proposed Kansei engineering (KE)-based approach.

The proposed application of a KE-based approach to dental healthscape design comprises six steps, which are illustrated in Figure 3 and detailed as follows.

*Step 1: Choice of design domain*

The dental healthscape was the design domain of this study. Individuals over 20 years old having the experience of receiving dental care services were the target population of this study.

*Step 2: Spanning the semantic space*

We established a Kansei vocabulary space corresponding to clients’ possible positive Kansei regarding dental services. The Kansei words were identified from dental books, magazines, and online reviews. The KJ method (i.e., affinity diagram) [67] was implemented to establish the Kansei vocabulary. In the KJ method, seven experts were invited: a literature professor, a human resources lecturer, a financial consultant, an online writer, a social worker, an advertising planner, and a dentist. The KJ method utilized expert discussion for removing inappropriate words and grouping words of similar meanings. Any words that did not fit into a group were left to one side, forming a group of their own. Finally, a word was picked from each group to represent that group, and to express clients’ specific emotions toward the dental service process. 

*Step 3: Spanning the property space*

To establish a space of design elements for the dental healthscape, we referred to an American Dental Association publication [68] and the clinical experience of dentists to select some common dental healthscape designs as examples. Hightower [65] conceptualized the perceived servicescape using three fundamental dimensions of the physical environment: (1) design factors, referring to the visible or tangible elements in a servicescape that stimulate perception directly; (2) ambience factors, including the background environmental elements that exist outside of immediate perception; and (3) social interaction factors, referring to the people involved in the servicescape. According to Hightower’s method [65], our dental healthscape design examples were deconstructed into their design elements. For example, the dentist’s attire is a factor of design, music is a factor of ambience, and the manner in which the dentist chats with clients is a factor of social interaction. Finally, various common attributes were set for each design element. For example, the element of dentist attire comprised three possible attributes: dark casual clothes, a conventional white physician gown, or a blue surgery gown. A specific scenario for the dental healthscape can be generated by combining all of the design elements, each of which is presented by one selected attribute.

*Step 4: Synthesis of the semantic space and property space*

This step involved synthesizing the semantic and property spaces to establish a link between them. One attribute of each element was selected and combined with those of other elements to generate a specific stimulus in KE. Each stimulus was a possible healthscape scenario for dental service. Various stimuli were generated using an orthogonal design method. Attributes of each element were incompatible and appeared with approximately even frequency across all generated stimuli. The semantic differential (SD) scale was used to evaluate the Kansei [69,70,71], producing a direct measurement of clients’ affective experience. Specifically, an SD questionnaire was presented with a randomly selected dental healthscape scenario (i.e., stimulus) using textual illustrations and photos, and respondents were asked to imagine being in the scenario and were then prompted to express their emotional response by rating each Kansei word. Each Kansei word was rated from 1 (*the healthscape scenario does not make me such Kansei*) to 5 (*the healthscape scenario strongly makes me such Kansei*). Additionally, respondents were asked to rate their revisit intention provoked by the stimulus from 1 (*weak intention*) to 5 (*strong intention*).

This synthesis of property space and semantic space required testing for validity. Exploratory factor analysis was used to validate the effectiveness of the space synthesis, focusing primarily on the adequacy of the Kansei words in the semantic space [66]. A Kansei word with significant load on at least one latent factor meant that the Kansei was indeed caused by the healthscape and should be retained. On the contrary, a Kansei word was removed only if it was with low loads on all latent factors. In such a case, we would return to Step 2 and update the semantic space.

*Step 5: Analysis of the relationship between the semantic and property spaces*

This step used partial least squares (PLS) [71,72] to analyze the questionnaire data and identify the relationships of Kansei with revisit intention and the design elements of dental healthscapes. PLS generalizes and combines features from principal component analysis and multiple regression and is often used to examine interrelationships among multiple independent and dependent variables, especially when the independent variables are numerous and highly collinear. In addition, variables are not required to follow a normal distribution for PLS. Prediction through PLS is achieved by extracting the orthogonal latent factors with the best predictive power from the independent variables [71,72,73]. 

Two relationship models were constructed:

(1) $Y_{revisit\;intention}$ = $f_{\left(client\;Kanseis\right)}$
(2) $Y_{Kansei}$ = $f_{\left(design\;elements\right)}$

In the first model, the strength of a client’s revisit intention is the dependent variable, and the client Kansei ratings are the independent variables. This model highlights the influence of each Kansei on clients’ willingness to revisit. In the second model, the rating of each Kansei is the dependent variable, and design elements are the independent variables. Through this model, the effect of each design element on each Kansei can be determined. 

*Step 6: Development of the design ideas*

Step 5 determined how the healthscape elements should be concerned and which attributes should be retained or avoided to help clients maintain positive emotions while receiving dental service and to increase their revisit intentions. This knowledge can be used to develop client-centered healthscapes.

## 3. Results

### 3.1. Semantic and Property Spaces

Twenty words were identified through the KJ method as central to the positive Kanseis of clients receiving dental service: *capable* (K1), *trustworthy* (K2), *hospitable* (K3), *tender* (K4), *steady* (K5), *friendly* (K6), *informed* (K7), *obliging* (K8), *helpful* (K9), *relieved* (K10), *comfortable* (K11), *encouraging* (K12), *calm* (K13), *clean* (K14), *cozy* (K15), *warm* (K16), *relaxed* (K17), *healthful* (K18), *thoughtful* (K19), and *hopeful* (K20).

With consideration given to the three dimensions of design, ambience, and social interaction [65], 17 design elements of the dental healthscape (E1–E17) were selected, each of which had two or three attributes, resulting in a total of 39 attributes (A1–A39), as displayed in the left column of Table 1.

### 3.2. Healthscape Stimuli for the Questionnaire Survey

Through an orthogonal design, 24 dental healthscape stimuli were created, as exhibited in the right column of Table 1. Each stimulus was formed by combining the 17 elements with a selected attribute—1 meant the stimulus included the attribute, whereas 0 meant it did not include the attribute.

### 3.3. Sampling and Data Collection

Individuals over 20 years old having the experience of dental treatment were the target population of this study. Participants were enrolled through convenience sampling and recruited from the Internet and social media. Prior to participating in the survey, the definition of the dental healthscape was explained, and the eligibility of the participants was confirmed. Overall, 600 participants, 25 for each stimulus, were surveyed during the period from July 2017 to May 2018. Each participant was asked to conduct Kansei evaluations on only one stimulus. Finally, 555 valid questionnaire responses (validity rate = 92.5%) were included in the subsequent analysis. The demographic characteristics of the participants are presented in Table 2.

### 3.4. Validity Testing

Table 3 shows the factor analysis results using principle component analysis with varimax rotation of factors to analyze the data from the 555 questionnaires. The Kaiser–Meyer–Olkin measure of sampling adequacy was 0.972, and the *p*-value of Bartlett’s test of sphericity was 0.000. Two factors with eigenvalues higher than 1 were extracted, explaining 73.258% of the total variance. The factor loading of each Kansei word to the factor to which it belonged was greater than 0.5, indicating that all 20 Kansei words were valid for the semantic space. Although some words (e.g., hopeful, relieved, obliging, helpful, tender, and friendly) had the problem of cross-loading, this only demonstrated that people’s emotions may not be explained by one factor alone and did not affect the appropriateness of using them for expressing client emotions. In addition, the Cronbach’s α value and composite reliability of each factor were both higher than 0.9, indicating internal consistency (reliability) in the Kansei evaluations. Factor 1 contained 11 Kansei words, most of which were related to the emotions caused by environment. Factor 2, which contained nine Kansei words, was mostly related to the emotions caused by dental staff.

### 3.5. Relationship between the Semantic Space and Property Space

Using the first relationship model ($Y_{revisit\;intention}$ = $f_{\left(client\;Kanseis\right)}$), the PLS results revealed that all 20 Kanseis had a positive effect on the dental revisit intention of clients (Bootstrap *t*-value > 2.57, *p* < 0.01). The five Kanseis most strongly related to revisit intention were *thoughtful* (K19), *hopeful* (K20), *tender* (K4), *comfortable* (K11), and *cozy* (K15), as listed in Table 4.

Using the second relationship model ($Y_{Kansei}$ = $f_{\left(design\;elements\right)}$), PLS identified 17 attributes that had significant effects on the Kanseis (Bootstrap *t*-value > 2.57, *p* < 0.01). Six attributes belonged to the dimension of design: “dentist wears blue surgery gown” (E3A7), “dentist’s name is shown” (E4A8), “dentist’s name is not shown” (E4A9), “using demo picture or sample for treatment explanation” (E5A10), “without demo pictures or samples” (E5A11), and “dentist wears a mouth mask only” (E6A14). Three attributes belonged to the dimension of ambience: “with music” (E11A24), “without music” (E11A25), and “without disinfection action in front of clients” (E12A28). Eight attributes belonged to the dimension of social interaction: “dentist chats with client” (E13A29), “dentist does not chat with client” (E13A30), “dentist tells client before any operation” (E14A31), “dentist does not tell client before any operation” (E14A32), “client opens mouth less than 20 minutes each time” (E15A33), “client opens mouth more than 20 minutes each time” (E15A34), “assistant is always at chair side” (E16A35), and “no assistant is next to the dental unit” (E16A37). The cross-loadings included in Table 5 quantify the effects of these significant attributes on each of the 20 Kanseis.

## 4. Discussion

**Figure 4:**
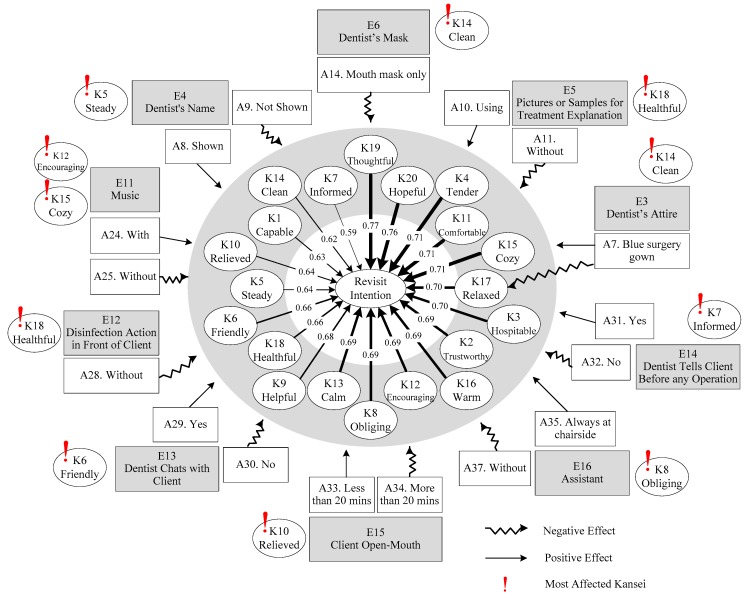
Healthscape element–Kansei–revisit intention relationship plot.

In this study, all 20 Kanseis had significant effects on the clients’ revisit intentions. This highlights the importance of maintaining positive Kansei in dental services. Studies have demonstrated that clients can benefit from regular oral care and immediate treatment more when the positive atmosphere of the dental healthscape reduces their fear and anxiety [27,28,74]. Also, this creates the elements of positive experience and physical, psychological, or emotional safe care for the building of green clinical care. To achieve positive emotions, 10 healthscape elements with 17 attributes had significant effects on the them. On the basis of the results shown in Table 4 and Table 5, Figure 4 visualizes the healthscape element–Kansei–revisit intention relationships which are then discussed in this section.

The five Kanseis had the strongest positive impacts on revisit intentions: *thoughtful*, *hopeful*, *tender*, *comfortable*, and *cozy*. *Thoughtful* was identified as the most noteworthy Kansei for clients involved in the dental healthscape. This emphasizes the importance of enhancing patient-centered care, in which a healthcare provider must understand the biopsychosocial context of patients and provide care in accordance with their cultural values, requirements, and preferences [75,76,77]. A thoughtful treatment plan and a process with clear treatment goals helps patients to feel more hopeful [78]. *Hopeful* was also a Kansei increasing clients’ revisit intention. *Hopeful* is an exceptional positive emotion that builds resilience and optimism [39]. A hopeful atmosphere motivates a client to participate actively in dental service. A study of orofacial pain suggested that a resilience-based hope intervention was beneficial for reducing pain sensitivity [79]. Previous research has indicated that patient perceptions of the physician’s compassion had a positive effect on healthcare [80]. Empathy is a critical dimension for measuring service quality [81]. Tenderness is a basic constituent of empathy and especially related to caregiving [82]. Linsher et al. [83] suggested that weakness evokes tender feelings even when the context is not immediate in nature. The present study determined that *tender* was one of the Kanseis most positively related to clients’ revisit intention. Comfort is a positive concept that can increase people’s health-seeking behavior [84]. Spake et al. [85] provided evidence that comfort has a substantial effect on consumer satisfaction, trust, and commitment with service providers. In this study, the *comfortable* Kansei increased clients’ revisiting intention. A *cozy* atmosphere, which was identified important in this analysis, helps to reduce stress and induce psychological well-being when people encounter an unfamiliar environment and feel anxious about an upcoming event [86]. To create specific Kansei for clients, Table 5 reveals the effects of healthscape elements in detail; these effects can serve as references for dental healthscape design.

Several results regarding the healthscape elements of the design dimension were noteworthy. Compared with the conventional white physician gown, a blue surgery gown was the most closely related to the *clean* Kansei; this result is different from results of previous studies [87,88]. In Taiwan, a blue surgery gown is often seen in the special examination room or intensive care unit and gives people an impression of a high degree of medical hygiene. Furthermore, displaying the dentist’s name helps clients identify the dentist. Having full access to the dentist’s information contributed the most to the clients’ *steady* Kansei. Similarly, medical demonstrations and sample models to patients have been identified as valuable tools in the healthscape [51,89]. This study confirmed these results and furthermore discovered that using demo pictures or samples for treatment explanation contributed highly to the *healthful* Kansei of clients. This finding may explain why people who were anxious about visiting dentists preferred knowing the causes and procedures of dental treatment by using cartoons or X-ray films rather than using pictures of real teeth [32]. “Dentist wears a mouth mask only” most negatively affected the *clean* Kansei. Conversely, “dentist wears a large face-mask” signaled good infection control [86].

Ambience is a critical concern of the design of the dental service process [68]. For example, playing music has been demonstrated to reduce the anxiety of people in waiting rooms [86,88,89,90]. Music also decreases blood plasma cortisol levels and reduces negative emotions [70]. This study confirmed that music in the healthscape creates positive emotions and determined that music mostly affected the *cozy* and *encouraging* Kansei. A study found that parents’ encouragement stimulated children’s engagement in healthy behavior [91]. Similarly, our study indicated that music stimulated a client’s dental visit intention. In addition, the attribute of “without disinfection action in front of clients” most negatively affected the *healthful* Kansei, indicating that deliberately allowing clients to observe disinfection actions is beneficial.

Several results regarding the healthscape elements of the social interaction dimension are discussed as follows. A client sitting in the dental chair rarely knows what will happen in the dental procedure. If the dentist does not provide an explanation, the client feels helpless [31,89,92]. The results of our study indicated that the dentist chatting with the client most affected the *friendly* Kansei, and telling the client before performing an operation most affected the *informed* Kansei. Clients tend to feel better if preparatory information is tailored and comprehensive [32,89]. We believe that these dentist–client interactions improve the dentist–client relationship, reduce the client’s anxiety about receiving dental services [32,89,92], and create positive social bonds between the client and service provider [81]. In particular, dialog between the dentist and client before the procedure, throughout the process, and even after care is an effective communication method. The attribute of “open mouth less than 20 minutes each time” strongly affected the *relieved* Kansei, as demonstrated in previous studies [74,88]. Clients were allowed to express their feelings, doubts, and thoughts to the dentist by raising a hand, which possibly alleviated the anxiety. No assistant next to the dental unit had the most negative effect on the *obliging* Kansei. An assistant helps the dentist control infection, assuage client anxiety, and ensure safety; thus, an assistant facilitates high-quality care [90]. Having an assistant chairside made clients feel *warm* and *obliging*, improving client satisfaction.

The design of the dental office and practice relates to the dentist’s brand [68]. By understanding the Kansei of clients, client-centered dental healthscape design can be implemented. A dental healthscape can be built that magnifies key positive Kanseis to eliminate anxiety, rejection, and other negative emotions in clients and increase their motivation to seek dental services. Importantly, this contributes to the effective and appropriate use of medical resources, reducing waste and harmful emissions which affect the environment.

## 5. Conclusions

Dentistry is highly energy- and resource-intensive with a significant environmental impact. Having the nature of service industries, dental service is a highly interactive experience between care providers and receivers [93]. Communication and participation influence client satisfaction and perceived value [94]. Enhancing client perceived value and motivating clients’ positive emotions in the medical process is the best way to promote preventive dental care, early treatment, and effective use of medical resources, which consequently reduces the harm to the environment in the long run, and contributes to green dentistry supply chains. 

Treatment is beneficial when clients voluntarily and honestly provide relevant information, including their current health status, expected outcomes, and affordable risks [42,43]. The willingness of clients to visit dentists affects their pursuit of preventive dental and periodontal care, early treatment, and regular oral examinations, affecting their physical health, psychological condition, and quality of life [95]. In Taiwan, the satisfactory health insurance system and high quality of medical care have overcome most barriers in accessing medical care. However, psychological barriers such as fear and anxiety remain.

Several methods for reducing dental anxiety have been proposed, including explanation of the treatment steps, administering drugs, and improving the clinical environment [32,74]. Research indicated that patients with anxiety prefer nonpharmacological intervention because of the low perceived medical risks [96]. Client-centered design of the healthscape is a method for avoiding pharmacological intervention. This is also a green practice that is important for reducing resource use and maintaining environmental sustainability. 

On the theoretical basis of the SOR model, this study applied KE to understand the effects of dental healthscape elements on clients’ Kansei and the subsequent effects on dental revisit intention. Kansei, inspired by Japanese philosophy and culture, illustrates the emotions and experiences of people in specific environments [59]. This study examined the design of client-centered dental healthscapes through KE to devise a space in which clients generate and maintain positive emotions when they visit. Positive emotions present new possibilities and provide clients with a wide range of thoughts and actions [39]. In addition, this study investigated the physical environment and client–dentist interactions of the dental healthscape. Consistent with the Gestalt framework [19,53], the findings of this study indicate that clients holistically evaluate dental visit experiences. Design factors (e.g., the dentist’s name is shown), ambience factors (e.g., music), and social interaction factors (e.g., the dentist chats with clients and tells them before performing any operation) were linked to the client Kansei and dental revisit intentions. These results provide references for healthscape design to help clients maintain positive emotional states during care and increase dental revisit intention, benefiting the promotion of preventive dental care, early treatment, and regular use of dental services.

This study makes several theoretical contributions. First, KE is an ergonomic methodology of affectional design for products and services. This study demonstrates its effectiveness in empirical studies of the SOR model. Second, little comprehensive research on the emotions of clients in the dental healthscape exists. However, understanding the effects of dental healthscape elements on clients’ Kansei and the subsequent effects on dental revisit intention is critical for promoting dental health. This study fills that particular research gap. Third, the clients’ Kansei and evaluations of visit experiences are stimulated by the healthscape holistically. Studying the effects of a single element without considering its interactions with others is insufficient. Clients’ experience is holistic, encompassing every contact with the healthscape [57]. This study examined the combination of all design elements of the healthscape, including the physical environment, service delivery process, and social interaction factors. This is the major advantage of using a KE-based method. Finally, this study provides a method for health service providers to build a client-centered care practice, which improves both health and quality of life. Compared with the sedative anesthesia method of using drugs, this study proposes the reduction of the clients’ anxiety state and the boosting of positive emotions by improving the environmental atmosphere of the dental healthscape, which is green.

Some limitations of this study indicate future research directions. First, in addition to healthscape, there are other internal and external factors of the dentistry supply chain that affect client value and green practices; however, they were not considered in this study. Second, textual illustrations and photos were used to present stimuli in the questionnaire. The use of these materials may have provided insufficient stimulation and weakened the formation of Kansei. Third, the clinical techniques of the dentists were not considered in this study; these potentially influence Kansei and thus should not be ignored. Finally, this study did not consider demographics; different groups of people have dissimilar needs.

## Figures and Tables

**Table 1:** Abbreviated table of property space and stimulus design.

Dimension	Element		Attribute		Stimulus							
					1	2	…	12	13	…	23	24
Design	E1	Outside-facing Windows	A1	Without	1	1	…	1	0	…	0	0
			A2	With	0	0	…	0	1	…	1	1
	E2	Dental Chair Unit	A3	Rod type	1	1	…	0	0	…	1	1
			A4	Cart type	0	0	…	1	1	…	0	0
	E3	Dentist’s Attire	A5	Dark casual clothes	1	0	…	0	1	…	0	0
			A6	Traditional white physician gown	0	1	…	1	0	…	1	0
			A7	Blue surgery gown	0	0	…	0	0	…	0	1
	E4	Dentist’s Name	A8	Shown	1	1	…	0	1	…	1	1
			A9	Not shown	0	0	…	1	0	…	0	0
	E5	Pictures or Samples for Treatment Explanation	A10	Using	1	1	…	1	1	…	1	1
			A11	Without	0	0	…	0	0	…	0	0
	E6	Dentist’s Mask	A12	Large facial mask	1	0	…	0	0	…	1	0
			A13	Safety goggles	0	1	…	0	0	…	0	1
			A14	Mouth mask only	0	0	…	1	1	…	0	0
Ambience	E7	Unit Partition	A15	No partition	1	0	…	0	1	…	0	0
			A16	Low partitions	0	1	…	0	0	…	1	0
			A17	High partitions	0	0	…	1	0	…	0	1
	E8	Color Tone	A18	Cool color (white, green, etc.)	1	1	…	0	1	…	0	0
			A19	Warm color (pink, hazel, etc.)	0	0	…	1	0	…	1	1
	E9	Placement of Instruments	A20	On metal tray	1	1	…	0	0	…	1	1
			A21	On disinfected paper	0	0	…	1	1	…	0	0
	E10	Cup for Gargle	A22	Disinfected metal cup	1	1	…	1	1	…	0	0
			A23	Disposable cup	0	0	…	0	0	…	1	1
	E11	Music	A24	With	1	1	…	1	0	…	1	1
			A25	Without	0	0	…	0	1	…	0	0
	E12	Disinfection Action in Front of Client	A26	Change devices’ wrap	1	0	…	1	0	…	0	1
			A27	Wipe devices with alcohol	0	1	…	0	0	…	0	0
			A28	Without	0	0	…	0	1	…	1	0
Social Interaction	E13	Dentist Chats with Client	A29	Yes	1	1	…	0	0	…	0	0
			A30	No	0	0	…	1	1	…	1	1
	E14	Dentist Tells Client Before any Operation	A31	Yes	1	1	…	0	1	…	0	0
			A32	No	0	0	…	1	0	…	1	1
	E15	Client Open-Mouth	A33	Less than 20 minutes each time	1	1	…	1	0	…	0	0
			A34	More than 20 minutes each time	0	0	…	0	1	…	1	1
	E16	Assistant	A35	Always at chairside	1	0	…	0	0	…	0	1
			A36	With sometimes	0	1	…	0	0	…	0	0
			A37	Without	0	0	…	1	1	…	1	0
	E17	Taking X-ray	A38	By dentist	1	0	…	1	0	…	1	0
			A39	By assistant	0	1	…	0	1	…	0	1

**Table 2:** Demographic characteristics of respondents (*N* = 555).

Characteristic		Frequency	Percentage (%)
Gender	Male	228	41.1
	Female	327	58.9
Age	≤30	91	16.4
	31–40	102	18.4
	41–50	107	19.3
	51–60	206	37.1
	≥61	49	8.8
Education	High school or below	69	12.4
	College	287	51.7
	Graduate or above	199	35.9
Occupation	Student	59	10.6
	Medical industry related	137	24.7
	Others	359	64.7
Frequency of visit dental clinic in recent year	More than 2 times	315	56.8
	Less than 2 times	240	43.2

**Table 3:** Factor analysis of Kansei evaluations.

Kansei	Factor Loading	
	Factor 1	Factor 2
K17 Relaxed	**0.853**	0.319
K13 Calm	**0.816**	0.351
K11 Comfortable	**0.806**	0.354
K16 Warm	**0.799**	0.382
K15 Cozy	**0.789**	0.399
K12 Encouraging	**0.745**	0.451
K19 Thoughtful	**0.723**	0.486
K14 Clean	**0.692**	0.391
K20 Hopeful	**0.683**	0.537
K18 Healthful	**0.667**	0.492
K10 Relieved	**0.593**	0.545
K1 Capable	0.242	**0.841**
K2 Trustworthy	0.337	**0.833**
K3 Hospitable	0.470	**0.738**
K5 Steady	0.400	**0.738**
K8 Obliging	0.547	**0.659**
K9 Helpful	0.558	**0.659**
K4 Tender	0.550	**0.658**
K7 Informed	0.388	**0.646**
K6 Friendly	0.561	**0.606**
Eigenvalues	8.054	6.653
Cumulative explained variances (%)	39.218	73.258
Cronbach α	0.964	0.949

**Table 4:** Cross-loading of significant Kansei word on revisit intention.

Kansei		Cross-Loading	Kansei		Cross-Loading
**K19**	Thoughtful	0.77	K20	Hopeful	0.76
**K4**	Tender	0.71	K11	Comfortable	0.71
**K15**	Cozy	0.71	K3	Hospitable	0.70
K17	Relaxed	0.70	K2	Trustworthy	0.69
K12	Encouraging	0.69	K8	Obliging	0.69
K16	Warm	0.69	K13	Calm	0.69
K9	Helpful	0.68	K6	Friendly	0.66
K18	Healthful	0.66	K5	Steady	0.64
K10	Relieved	0.64	K1	Capable	0.63
K14	Clean	0.62	K7	Informed	0.59

**Table 5:** Cross-loading of significant healthscape attributes on Kansei (*p* < 0.01).

Kansei		Design						Ambience			Social Interaction							
		E3	E4		E5		E6	E11		E12	E13		E14		E15		E16	
		Attire	Name		Demo		Mask	Music		Disinfection	Chat		Tell		Open-Mouth		Assistant	
		A7	A8	A9	A10	A11	A14	A24	A25	A28	A29	A30	A31	A32	A33	A34	A35	A37
K19	Thoughtful	0.09	0.10	−0.10	0.12	−0.12	−0.10	0.17	−0.17	−0.08	0.23	−0.23	0.21	−0.21	0.07	−0.07	0.14	−0.21
K20	Hopeful	0.08	0.09	−0.09	0.11	−0.11	−0.11	0.19	−0.19	−0.09	0.22	−0.22	0.21	−0.21	0.05	−0.05	0.13	−0.19
K4	Tender	0.05	0.10	−0.10	0.07	−0.07	−0.07	0.15	−0.15	−0.07	0.24	−0.24	0.21	−0.21	0.12	−0.12	0.12	−0.18
K11	Comfortable	0.06	0.07	−0.07	0.09	−0.09	−0.08	0.16	−0.16	−0.06	0.21	−0.21	0.19	−0.19	0.12	−0.12	0.11	−0.14
K15	Cozy	0.09	0.07	−0.07	0.13	−0.13	−0.08	**0.22**	**−0.22**	−0.07	0.23	−0.23	0.17	−0.17	0.10	−0.10	0.12	−0.16
K17	Relaxed	-0.02	0.08	−0.08	0.07	−0.07	−0.11	0.20	−0.20	−0.03	0.25	−0.25	0.22	−0.22	0.10	−0.10	0.15	−0.18
K3	Hospitable	0.10	0.12	−0.12	0.08	−0.08	−0.11	0.15	−0.15	−0.07	0.21	−0.21	0.21	−0.21	0.13	−0.13	0.15	−0.20
K2	Trustworthy	0.14	0.12	−0.12	0.12	−0.12	−0.08	0.13	−0.13	−0.11	0.22	−0.22	0.19	−0.19	0.07	−0.07	0.12	−0.21
K16	Warm	0.08	0.08	−0.08	0.12	−0.12	−0.10	0.21	−0.21	−0.09	0.23	−0.23	0.19	−0.19	0.11	−0.11	**0.16**	−0.20
K12	Encouraging	0.02	0.09	−0.09	0.07	−0.07	−0.09	**0.22**	**−0.22**	−0.06	0.29	−0.29	0.28	−0.28	0.13	−0.13	0.13	−0.16
K8	Obliging	0.05	0.11	−0.11	0.05	−0.05	−0.08	0.10	−0.10	−0.01	0.29	−0.29	0.33	−0.33	0.09	−0.09	**0.16**	**−0.22**
K13	Calm	0.07	0.10	−0.10	0.07	−0.07	−0.10	0.14	−0.14	−0.04	0.23	−0.23	0.23	−0.23	0.11	−0.11	0.10	−0.16
K9	Helpful	0.09	0.10	−0.10	0.07	−0.07	−0.06	0.12	−0.12	−0.11	0.25	−0.25	0.27	−0.27	0.12	−0.12	0.14	−0.18
K18	Healthful	0.12	0.03	−0.03	**0.14**	**−0.14**	−0.10	0.18	−0.18	**−0.15**	0.15	−0.15	0.17	−0.17	0.09	−0.09	0.14	−0.17
K6	Friendly	0.04	0.11	−0.11	0.10	−0.10	−0.09	0.15	−0.15	−0.04	**0.35**	**−0.35**	0.21	−0.21	0.08	−0.08	0.08	−0.21
K5	Steady	0.14	**0.16**	**−0.16**	0.07	−0.07	−0.11	0.16	−0.16	−0.12	0.15	−0.15	0.15	−0.15	0.09	−0.09	0.09	−0.07
K10	Relieved	0.08	0.07	−0.07	0.07	−0.07	−0.05	0.12	−0.12	−0.09	0.20	−0.20	0.15	−0.15	**0.14**	**−0.14**	0.11	−0.13
K1	Capable	0.13	0.12	−0.12	0.13	−0.13	−0.11	0.14	−0.14	−0.08	0.18	−0.18	0.14	−0.14	0.08	−0.08	0.08	−0.17
K14	Clean	**0.15**	0.11	−0.11	0.11	−0.11	**−0.12**	0.21	−0.21	−0.10	0.26	−0.26	0.12	−0.12	0.02	−0.02	0.11	−0.17
K7	Informed	0.02	0.10	−0.10	0.01	−0.01	−0.04	0.01	−0.01	0.02	0.19	−0.19	**0.52**	**−0.52**	0.07	−0.07	0.14	−0.19
